# Periodontitis-level butyrate-induced ferroptosis in periodontal ligament fibroblasts by activation of ferritinophagy

**DOI:** 10.1038/s41420-020-00356-1

**Published:** 2020-11-10

**Authors:** Yunhe Zhao, Jiao Li, Wei Guo, Houxuan Li, Lang Lei

**Affiliations:** 1 Nanjing Stomatological Hospital, Medical School of Nanjing University, 210008 Nanjing, China; 2 Central Laboratory of Stomatology, Nanjing Stomatological Hospital, Medical School of Nanjing University, 210008 Nanjing, China

**Keywords:** Cell death, Dental diseases

## Abstract

Loss of periodontal ligament fibroblasts (PDLFs) is one critical issue for regenerating lost periodontal tissues. A wide variety of regulated cell death pathways, such as apoptosis, pyroptosis, and necroptosis have been proposed in the periodontitis development. The aim of the present study was to explore whether long-term periodontitis-level butyrate may trigger ferroptosis, a newly characterized iron-dependent regulated cell death in PDLFs. Here, we showed that long-term treatment of butyrate, an important short-chain fatty acid in the periodontal pocket, induces the cargo receptor nuclear receptor coactivator 4 (NCOA4)-mediated ferritinophagy and ferroptosis in PDLFs. Butyrate-induced iron accumulation, reactive oxygen species (ROS) generation, glutathione depletion and lipid peroxidation in PDLFs, and the butyrate-induced ferroptosis can be blocked by the lipid peroxide scavenger ferrostatin-1. The NCOA4-mediated ferritinophagy is dependent on p38/hypoxia inducible factor-1α (HIF-1α) pathway activation as well as Bromodomain-containing protein (BRD) 4 and cyclin-dependent kinase 9 (CDK9) coordination. These lines of evidence provide a new mechanistic insight into the mechanism of loss of PDLFs during periodontitis development, showing that periodontitis-level butyrate disrupted iron homeostasis by activation of NCOA4-mediated ferritinophagy, leading to ferroptosis in PDLFs.

## Introduction

Periodontitis, an inflammatory disease that affects the supporting tissues of the teeth, is initiated by the dysbiosis of dental biofilms in the periodontal milieu^1^. Persistent growth and proliferation of anaerobic periodontal pathogens, such as *Porphyromonas gingivalis*, *Fusobacterium nucleatum*, *Tannerella forsythia*, and *Prevotella intermedia*, may produce large amounts of short-chain fatty acids (SCFAs), including succinic acid, lactic acid, acetic acid, and butyrate. Elevated levels of butyrate (up to 16 mM) have been found in the gingival crevicular fluids (GCFs) of patients with chronic and aggressive periodontitis, with its concentration strongly correlated with pocket depth^2–6^.

Butyrate, which can also be produced by commensal bacteria in the colonic lumen from fermentation of dietary fibers, can be utilized by intestinal epithelial cells to protect gut epithelium integrity and modulate the intestinal immune system^7^. In contrast, periodontitis-level butyrate plays a detrimental role in the periodontal tissues. It may inhibit fibroblast cell growth and proliferation, elicit oxidative stress as well as endoplasmic reticulum stress^8^, pyroptosis^9^, and apoptosis^10^. Although the periodontal ligament fibroblasts (PDLFs) can maintain the homeostasis of periodontal ligament through its proliferation and differentiation, persistent detrimental stimuli may lead to irreversible loss of the resident PDLFs.

To respond to the drastic changes in the extracellular environment, cells in the periodontal niche may undergo inflammatory responses or cell death to alert the immune defense system^11^. In addition to the non-inflammatory apoptosis, several types of regulated cell death (RCD) have been well recognized, such as apoptosis, pyroptosis, NETosis, and necroptosis. Apoptosis is reduced in diseased periodontal tissues^12^, while pyroptosis and necroptosis are increased in inflamed periodontal tissues^9,13^.

Ferroptosis is a recently recognized iron-dependent and caspase-independent RCD, which is characterized by accumulation of iron-dependent reactive oxygen species (ROS), leading to excessive lipid peroxidation and cell death. In nature, ferroptosis is defined as a metabolic dysfunction involving disturbed intracellular glutaminolysis and disrupted glutathione peroxidase 4 (GPX4), resulting in inadequate reduction of ROS and peroxidized lipid^14^. Iron plays a pivotal role in the process of ferroptosis. Normally, intracellular iron is mostly stored in the ferritin. Ferritin-bound iron can be presented to autophagosomes by cargo receptor nuclear receptor coactivator 4 (NCOA4), and this process is named as ferritinophagy^15^. Irons released from ferritin as its free form enter the transient labile iron pool (LIP) for Fenton reaction, resulting in elevated generation of ROS.

Ferroptosis has been implicated in multiple pathologic conditions such as tumorigenesis, ischemia–reperfusion injury^16^, degenerative diseases^17^, and stroke^18^. In addition, physical stress, such as cigarette smoke exposure^19^ and cold stress^20^, can induce ferroptosis in cancer cells, epithelial cells, and vascular smooth muscle cells. However, whether the secondary metabolite butyrate from periodontal pathogens can induce ferroptosis in the PDLFs and its role in the periodontitis progress has never been reported. Hereby we discovered that periodontitis-level sodium butyrate treatment-induced ferroptosis in PDLFs, and the onset of ferroptosis is closely correlated with activation of NCOA4-mediated ferritinophagy; moreover, the p38/hypoxia inducible factor-1α (HIF-1α) pathway activation as well as bromodomain-containing protein (BRD) 4 and cyclin-dependent kinase 9 (CDK9) coordination regulated onset of ferritinophagy and ferroptosis.

## Results

### Butyrate-induced multiple types of RCD in PDLFs

**Fig. 1:**
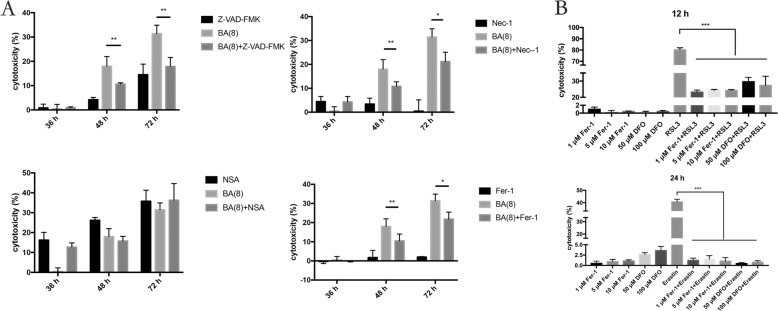
Butyrate-induced multiple types of regulated cell death in PDLFs. **A** PDLFs were pretreated with a pan-caspase inhibitor (10 μM Z-VAD-FMK), a RIP1 inhibitor (20 μM necrostatin-1), an MLKL inhibitor (10 μM necrosulfonamide) or lipid peroxide scavengers (5 μM ferrostatin-1) for 2 h, and then cells were stimulated with butyrate (8 mM) for 48 h. **B** PDLFs were incubated with 1, 5, or 10 μM ferrostatin-1 and 50, 100 μM DFO for 2 h, and then treated with 20 μM RSL3 for 12 h or 20 μM Erastin for 24 h before pretreatment with 1, 5, or 10 μM ferrostatin-1 and 50, 100 μM DFO for 2 h. Cell death was assessed by LDH assay. Mean ± SD, *n* = 3. *p < 0.05, **p < 0.01, ***p < 0.001 vs. control group.

Periodontitis-level butyrate of 8 μM did not trigger significant cell death at early time of treatment before 24 h. No significant release of LDH from PDLFs can be detected even at 36 h, while obvious cell death could be observed at 48 and 72 h. NSA failed to inhibit cell death in PDLFs after butyrate stimulation, while Z-VAD-FMK and Necrostatin-1 (Nec-1) significantly reduced cell death by butyrate stimulation. As Nec-1 can both block necroptosis and apoptosis, the effect of cell death inhibition by Nec-1 was apoptosis in the butyrate-treated PDLFs. Moreover, we explored whether ferrostatin-1 (Fer-1) can reduce cell death. Pretreatment with Fer-1 significantly reduced cell death 48 and 72 h after butyrate stimulation, indicating that ferroptosis may participate in the process of cell death (Fig. 1A).

Furthermore, we explored whether ferroptosis can be induced in PDLFs by specific agents, RSL3 (a specific inhibitor of GPX4) and Erastin (a specific inhibitor of System Xc—on the cell membrane). RSL3 induced ~80% of cells to undergo death after 12 h incubation in PDLFs, while Erastin triggered around 40% of cells to decease after 24 h. Fer-1 significantly blocked cell death induced by RSL3 and Erastin (Fig. 1B).

### Butyrate-induced ROS production and lipid peroxidation

**Fig. 2:**
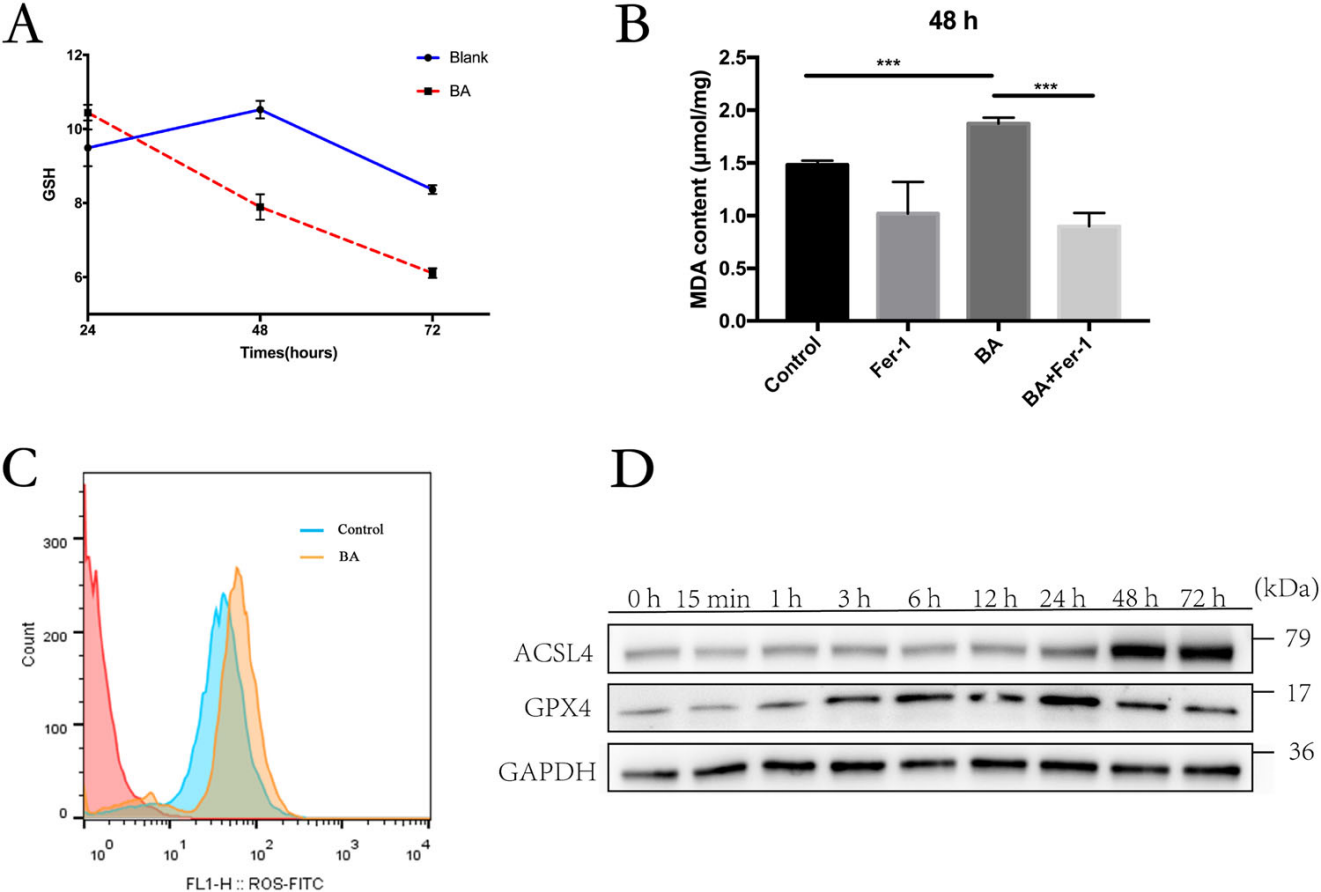
Butyrate-induced ROS production and lipid peroxidation. **A** Changes of the glutathione redox status after 24, 48, and 72 h butyrate treatment. **B** Analysis of malondialdehyde (MDA) generation and lipid peroxidation after butyrate incubation (8 mM) in PDLFs at 48 h after pretreatment with lipid peroxide scavenger (5 μM ferrostatin-1). **C** Intracellular ROS was detected by flow cytometry 24 h after butyrate treatment. **D** Protein levels of indicated genes were determined by Western blot. Mean ± SD, *n* = 3. *p < 0.05, **p < 0.01, ***p < 0.001 vs. control group.

Ferroptosis is a ROS-dependent cell death, and lipid peroxidation by excessive intracellular ROS plays a crucial role in its onset^21^; we then investigated whether butyrate induce excessive ROS generation in PDLFs. Long-term treatment with butyrate significantly reduced the level of GSH, which can be utilized by GPX4 as electron donors to reduce the intracellular ROS (Fig. 2A). Membrane polyunsaturated lipids are liable to oxidation by excessive ROS in the cytosol, leading to the generation of end products such as MDA. MDA levels were increased by the butyrate treatment in PDLFs (Fig. 2A). Following decreased levels of intracellular GSH, ROS level was significantly increased in butyrate-treated PDLFs (Fig. 2C). Furthermore, GPX4 expression after butyrate treatment in PDLFs rose as early as 1 h, climbed to highest at 24 h and dropped at 48 and 72 h. Moreover, butyrate treatment enhanced expression of acyl-CoA synthetase long-chain family member 4 (ACSL4), which enriches cellular membranes with long polyunsaturated ω6 fatty acids and predispose cells to undergo lipid peroxidation (Fig. 2D). Therefore, long-term treatment with butyrate caused excessive accumulation of ROS, leading to depleted GSH, reduced GPX4 and enhanced lipid peroxidation in PDLFs.

### Butyrate-induced iron accumulation is dependent on ferritinophagy in PDLFs

**Fig. 3:**
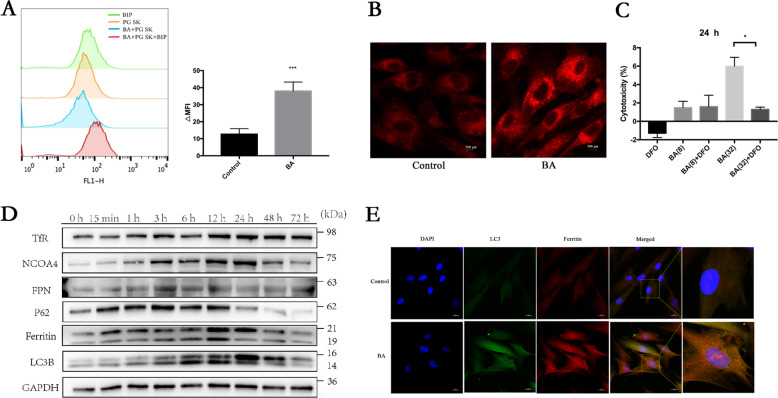
Butyrate-induced iron accumulation is dependent on ferritinophagy in PDLFs. **A** Labile iron pool was measured using PG SK method in control or butyrate (8 mM)-treated PDLFs. **B** Intracellular Fe^2+^ in control or butyrate (8 mM)-treated PDLFs were detected by FerroOrange. **C** PDLFs were pretreated with an iron chelator, deferoxamine (100 μM), for 2 h, and treated with butyrate (32 mM) for 36 h, then cell death was detected by LDH assay. **D** TfR, NCOA4, FPN, ferritin, P62, LC3B, and GAPDH expression in response to butyrate (8 mM) were assessed by Western blot. **E** Representative images of colocalization of ferritin (red) with LC3-GFP (green) in butyrate-treated PDLFs at 12 h. Scale bars = 20 μm.

Iron plays a key role in ferroptosis by generating excessive ROS by the Fenton reaction^22,23^. Fe^3+^ enters the cell through the TfR, and is reduced to Fe^2+^ by the STEAP3 metalloreductase in the endosome, and the free Fe^2+^ in the cell is stored in the ferritin. In order to investigate the characteristics of LIP in butyrate-treated PDLFs, free iron levels were monitored by fluorescence analysis. After incubation with butyrate (8 mM) for 12 h, the mean fluorescence intensity of PG SK decreased, which indicated a significant increase in the intracellular labile iron levels within the PDLFs (Fig. 3A). In addition, we detected levels of free iron by confocal microscopy observing significant elevation in free iron levels (Fig. 3B). We next explored whether reducing intracellular iron levels can reduce cell death. By chelating intracellular iron that is indispensable for cell survival, DFO itself may inhibit cell proliferation, induce cell cycle arrest, and promote cell apoptosis^24^. Although iron chelates deferoxamine (DFO) (Sigma, USA) failed to reduce LDH release in butyrate (8 mM)-treated PDLFs, DFO significantly mitigated cell death in butyrate (32 mM)-treated PDLFs (Fig. 3C).

Most iron was stored in ferritin, and autophagic degradation of ferritin, a process called ferritinophagy is activated once host cells need free iron for biological activities^23^. This new type of autophagy relies on a selective cargo receptor, NCOA4, which transfers ferritin to autophagosomes. The protein levels of NCOA4, ferritin, FPN, and TfR were remarkably elevated following butyrate treatment (Fig. 3D). Similarly, the protein level of LC3B and P62 was significantly altered in PDLFs with butyrate treatment (Fig. 3D). Immunofluorescence staining of butyrate-treated PDLFs showed that both ferritin and LC3 GFP expression were enhanced and colocalization of ferritin with LC3-GFP was detected, indicating degradation of ferritin in the autophagasome (Fig. 3E).

### Butyrate-induced ferroptosis was dependent on HIF-1a/p38 pathway activation and CDK9/BRD4 coordination

**Fig. 4:**
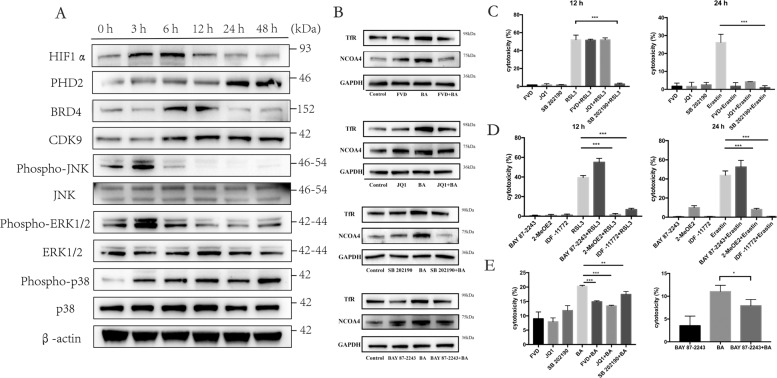
Butyrate-induced ferroptosis was dependent on HIF-1a/p38 pathway activation and CDK9/BRD4 coordination. **A** Time course of HIF1α, PHD2, BRD4, CDK9, phospho-JNK, JNK, phosphor-ERK1/2, ERK1/2, phospho-p38, p38, and β-actin expression in response to butyrate (8 mM). **B** Effects of CDK9 inhibitor (40 nM FVD), BRD4 inhibitor (250 nM JQ1), p38 inhibitor (10 μM SB 202190), and HIF1α inhibitor (5 nM BAY 87-2243) on NCOA4 and TfR at 24 h. **C**–**E** Cell death after treatment with RSL3 (20 μM) for 12 h, Erastin (20 μM) for a 24 h or butyrate (8 mM) for 48 h after preincubation with CDK9 inhibitor (40 nM FVD), BRD4 inhibitor (250 nM JQ1), p38 inhibitor (10 μM SB 202190), and HIF1α inhibitor (5 nM BAY 87-2243, 5 μM 2-MeOE2, 10 μM IDF-11772) for 2 h. Mean ± SD, *n* = 3. *p < 0.05, **p < 0.01, ***p < 0.001 vs. control group.

Mitogen-activated protein kinases (MAPK) are phosphorylated and activated by a plethora of stimuli, and the relative intensity and duration of their activation has been proposed to determine survival or death decisions made by cells. p38 activation has been reported to participate in cold stress-induced ferroptosis^20^. Firstly, we observed that butyrate initiated distinct phosphorylation signals in all three MAPKs in the PDLFs. Elevation of phosphorylated-JNK and phosphorylated-ERK1/2 can be observed at 3 h, while levels of phosphorylated-p38 remained elevated from 3 to 48 h (Fig. 4A). HIF-1α is one prominent transcription factor participating cellular metabolism, and butyrate recently has been reported to activate HIF-1α^25^. Elevation of HIF-1α was observed at 3 and 6 h after butyrate treatment, while increase in the prolyl hydroxylase domain 2 (PHD2) was detected at 24 and 48 h. Coordination of the complex of CDK9 and BRD 4 promotes active transcription elongation. Butyrate treatment increased CDK9/BRD4 expression at 3 and 6 h (Fig. 4A).

We next explored whether p38 and HIF-1α pathway activation participated in the initiation of ferroptosis, and investigated whether CDK9 and BRD4-mediated transcription elongation participated in the activation of ferroptosis. CDK9 inhibition by flavopiridol, BRD4 inhibition by JQ1, p38 inhibition by SB202190, HIF-1α inhibition by BAY-87-2243 reduced the level of NCOA4 and TfR after BA stimulation (Fig. 4B). Then, we used the cell-permeable ferroptosis activator Erastin and RSL3 to induce specific ferroptosis. PDLFs were pretreated with JQ1, flavopiridol, SB202190, and BAY 87-2243/2-MeOE2/IDF-11772 (inhibitors of HIF-1α) for 2 h. Blocking of p38, BRD4, and CDK9 significantly reduced RSL3 and Erastin-induced ferroptotic cell death. 2-MeOE2 and IDF-11772 significantly inhibited RSL3 and Erastin-induced cell death, while BAY 87-2243 promoted RSL3 and Erastin-induced cell death (Fig. 4C, D). In addition, CDK9 inhibition by flavopiridol, BRD4 inhibition by JQ1, p38 inhibition by SB202190, HIF-1α inhibition by BAY-87-2243 reduced the level of lactate dehydrogenase (LDH) after BA stimulation (Fig. 4E).

### HIF-1α–CDK9/BRD4 interaction promoted NCOA4-mediated ferritinophagy

**Fig. 5:**
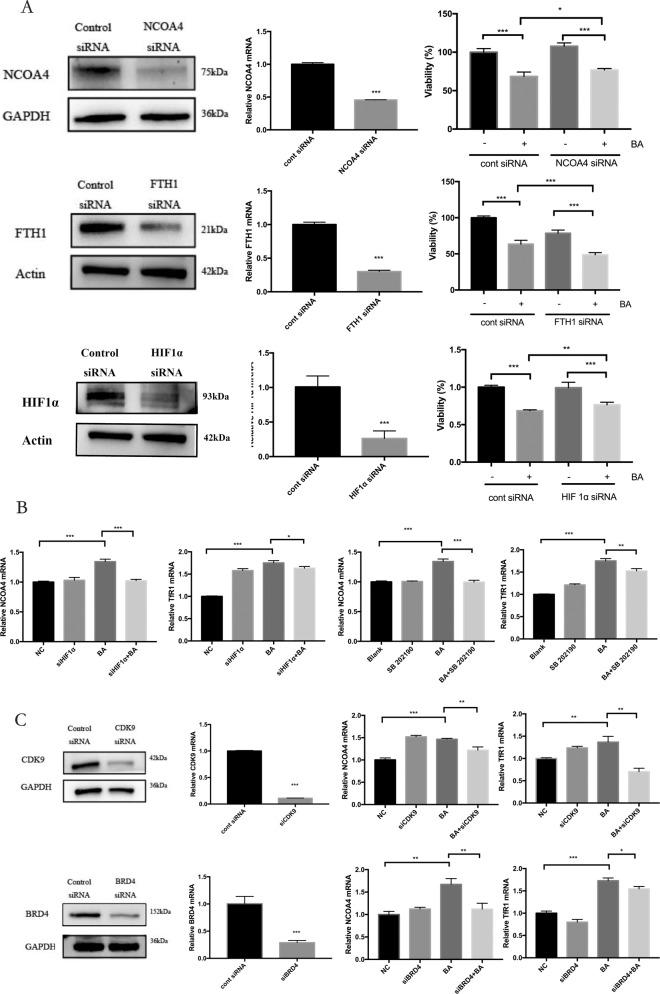
HIF-1α–CDK9/BRD4 interaction promoted NCOA4-mediated ferritinophagy. **A** PDLFs were transfected with control siRNA, NCOA4 siRNA, FTH1 siRNA, or HIF1α siRNA 36 h before butyrate (8 mM) treatment, cell viability was determined by CCK8 assay (*n* = 3). **B**, **C** PDLFs with CDK9 siRNA, BRD4 siRNA, HIF1α siRNA, or p38 inhibitor (10 μM SB 202190) were treated with butyrate (32 mM) for 24 h. Levels of mRNA in cells were determined by qPCR. Mean ± SD, *n* = 3. *p < 0.05, **p < 0.01, ***p < 0.001 vs. control group.

To further elucidate the molecular mechanisms for upregulated ferritinophagy in butyrate-induced cell death in PDLFs, we then focused on whether HIF-1α-mediated gene transcription was ferritinophagy-dependent. First, we investigated effects of siRNA-mediated knockdown of NCOA4, FTH1, and HIF-1α. NCOA4 and HIF-1α knockdown promoted the cell viability in butyrate-treated PDLFs (CCK8 assay), while FTH1 gene interference significantly reduced the cell viability in butyrate-stimulated PDLFs (Fig. 5A). We then analyzed whether HIF-1α affect NCOA4 and TfR1 transcription. HIF-1α knockdown significantly reduced butyrate-induced NCOA4 and TfR1 mRNA transcription; similarly, p38 inhibitor SB202190 decreased NCOA4 and TfR1 transcription (Fig. 5B). We further analyzed the importance of CDK9/BRD4 coordination in the butyrate-induced iron metabolism. CDK9 gene interference significantly reduced butyrate-induced NCOA4 and TfR1 transcription, while BRD4 knockdown reduced NCOA4 but not TfR1 transcription (Fig. 5C).

## Discussion

Periodontal homeostasis is maintained by a dynamic balance between cell death and survival of resident cells in the periodontal niche. We previously reported that necroptosis participated in the *P. gingivalis*-triggered cell death in PDLFs^26^, and the onset of necroptosis is dependent on CDK9 activation^13^. In our present study, we further demonstrated that NCOA4-mediated ferritinophagy and ferroptosis participated in the periodontitis-level butyrate-induced cell death; moreover, we further demonstrated that HIF-1α pathway activation and CDK9/BRD4 transcription modulation mediated butyrate-triggered NCOA4 expression in PDLFs.

Although NCOA4-mediated ferritinophagy has been implicated in chronic obstructive pulmonary disease, stroke, and ischemia-reperfusion injuries^21^, the molecular mechanisms modulating ferritinophagy and ferroptosis remain obscure. The cigarette smoke induced ferroptosis in bronchial epithelial cells^19,27^ and vascular smooth muscle cells^28^, and NCOA4-mediated ferritinophagy promoted ROS accumulation in epithelial cells^27^. Continuous severe cold stress-induced ferroptosisis relied on the apoptosis signal-regulating kinase 1 (ASK1)-p38 MAPK activation^20^, which is also involved in Erastin-induced ferroptosis^29^. Similarly, our experiment demonstrated that butyrate-induced ferritinophagy was dependent on p38 activation; moreover, we observed for the first time that HIF-1α activation contributed to ferroptosis. HIF-1α activation has also been observed in butyrate-treated mice to protect clostridium difficile-induced colitis^25^. How butyrate treatment can induce HIF-1α stabilization is not clear, and one possible explanation for HIF-1α stabilization is the effect of butyrate on the succinate dehydrogenase (SDH), which oxidizes succinate into fumarate, as a histone deacetylase (HDAC) inhibitor^30^. Compromised SDH activity may induce succinate accumulation, which dampens activity of dioxygenase, such as prolyl hydroxylases (PHDs), leading to HIF-1α stabilization^31^. Indeed, *P. gingivalis* infection in PDLFs has triggered metabolic reprogramming from oxidative phosphorylation to glycolysis with succinate accumulation, PHD2 suppression, and HIF-1α stabilization^32^.

The recruitment and binding of RNA Polymerase II with various transcription factors, such as p38 and HIF-1α, onto transcription start sites (TSS) is an important mechanism for modulating the expression of a myriad of target genes^33^. The coordination of BRD4 and CDK9 at TSS facilitates the transition from transcription pausing to elongation. We observed that CDK9 and BRD4 inhibition reduced butyrate-triggered NCOA4 expression, decreased Erastin, RSL3 and butyrate-induced cell death; in contrast, BRD4 inhibition by JQ1 triggered ferritinophagy and ferroptosis in cancer cells^34^. These data reveal that host cells can dynamically and intricately regulate cell survival and death in a stimuli-dependent manner.

The term ferroptosis was coined in 2012 by Dixon et al. to describe the form of cell death induced by the small molecule Erastin^35^. In general, ferroptosis should be suppressed by both an iron chelator (e.g., DFO) and a lipophilic antioxidant (e.g., ferrostatin and liproxstatin), and should involve the accumulation of lipid hydroperoxides^27^. Such features are all observed in our experiment, and we also observed dampened GPx4 expression but elevated ASL4 expression, which further suggest the onset of ferroptosis. Since butyrate can trigger apoptosis, pyroptosis, and autophagic cell death in gingival epithelial cells and fibroblasts^9,36,37^. Moreover, butyrate rather than LPS compromised the gingival epithelial barrier by triggering pyroptosis^9^. We observed that butyrate-induced cell death in PDLFs can be inhibited by pan-caspase inhibitor, Z-VAD-FMK, and Nec-1, indicating that butyrate may induce several intertwined pathways in PDLFs. Although our data suggest that butyrate may trigger ferroptosis in PDLFs, a GPx4-deficient animal model should be further used to explore the role of ferroptosis in the periodontitis development.

Iron is indispensable for the survival of both bacterium and vertebrate cells^1^. Host cells can also utilize iron to generate ROS to clear microbials and promote cell survival; however, excessive free iron buildup in the cytosol may promote overdue ROS generation, leading to cell death. Although long-term butyrate treatment triggered onset of ferritinophagy and generation of ROS in PDLFs, the pathological role of ferritinophagy should further be explored. For example, whether *P. gingivalis* can hijack irons release from ferritin to facilitate its growth should be investigated. Indeed, uropathogenic *Escherichia coli* manipulate the ferritinophagy to facilitate its persistence in bladder epithelial cells^15^.

In conclusion, our present data demonstrated that butyrate-induced ferroptosis may contribute to the loss of PDLFs during periodontitis development, and the onset of ferroptosis involves p38/HIF-1α activation and BRD4/CDK9 coordination.

## Materials and methods

### Cell culture

The primary human PDLFs were collected from 10 to 18-year-old patients who needed orthodontic extraction of healthy premolars. Written informed consent was obtained from each donor. The protocol for collecting gingival samples was approved by the Medical Ethics Committee of Nanjing Stomatological Hospital, Medical School of Nanjing University, and the ethics approval number was 2016NL-010(KS). All experiments were performed in accordance with relevant guidelines and regulations. Participants were informed of the purpose of experiments and provided informed consent. PDLFs were cultured as we previously described^32^. Briefly, the tissue explants obtained from the middle third of the root were cultured in DMEM (Gibco, USA) with 10% fetal bovine serum (Gibco, Australia) and 1% penicillin/streptomycin solution (Thermo Fisher Scientific, USA). Cells at the third to sixth passages were used in the experiment.

Nec-1 (Selleckchem, USA), necrosulfonamide (NSA) (Enzo Life Sciences, USA) were utilized to block RIPK1, RIPK3, and MLKL, respectively. Z-VAD-FMK (Selleckchem, USA), a pan-caspase inhibitor, and Fer-1 (Selleckchem, USA), a lipid peroxide scavenger. The PDLFs were pretreated with these inhibitors for 2 h and then stimulated with sodium butyrate for indicated times. To induce specific ferroptosis, PDLFs were also treated with RSL3 (Selleckchem, USA) and Erastin (Selleckchem, USA) for 24 h.

### Cytotoxicity assay

PDLFs were seeded into 96-well plates. Cell death rate was measured using the LDH-Cytotoxic Test (Promega, USA) basically following the manufacturer’s instruction. The absorbance value of DMEM medium served as benchmark, and the absorbance value of completely lysed cells was regarded as the maximal LDH release. The optical absorbance at 490 nm was measured at 490 nm using a Spectra Max M3 (Molecular Devices, Sunnyvale, CA, USA). Cell viability was measured using a Cell Counting Kit-8 (Biosharp, China) following the manufacturer’s instruction.

### Small interfering RNA (siRNA) transfection

Envirus^TM^ (Engreen Biosystem, China) was used to transfect PDLFs with small interfering RNA targeting CDK9, BRD4 or a control siRNA with no target (GenePharma, China). siRNA targeting NCOA4, FTH1, HIF-1α, and negative control siRNAs were purchased from PPL (Public Protein/Plasmid Library, China). The efficacy of knockdown was analyzed by qPCR 48 h and Western blot 72 h after transfection.

### Isolation of RNA and quantitative PCR

Total RNA was extracted using a RNAiso Plus kit (Tiangen, China) and measured using a Nanodrop (Thermo Fisher Scientific, USA). Real-time PCR was performed using SYBR Green Master MIX (ABI, USA). The β-actin gene was used as an internal control. The PCR primer sequences were as follows: FTH1 5′-CCCCCATTTGTGTGACTTCAT-3′ and 3′-GCCCGAGGCTTAGCTTTCATT-5′; NCOA4 5′-GAGGTGTAGTGATGCACGGAG-3′ and 3′-GACGGCTTATGCAACTGTGAA-5′; SLC40A1 5′-CTACTTGGGGAGATCGGATGT-3′ and 3′-CTGGGCCACTTTAAGTCTAGC-5′; TfR1 5′-ACCATTGTCATATACCCGGTTCA-3′ and 3′-CCAATAGCCCAAGTAGCCAATCAT-5′; GAPDH 5′-GGAGCGAGATCCCTCCAAAAT-3′ and 3′-GGCTGTTGTCATACTTCTCATGG-5′.

### Western blotting

Western blotting was performed as we described before^13^. Briefly, cells were washed with PBS and lysed. The concentration of total proteins was determined using a Nanodrop (Thermo Fisher, USA). Proteins were separated by 12% Bis–Tris Plus gels (Genscript, China), transferred to PVDF membrane (Millipore, USA), and blocked for 1 h at room temperature and incubated with primary antibodies, anti-transferrin receptor (TfR) (1:1000; ab214039, Abcam, USA), anti-NCOA4 (1:1000; A5695, ABclonal, China), anti-ferritin (1:1000; ab65080, Abcam), anti-ferroportin (FPN) (1:1000; ab78066, Abcam), anti-LC3B (1:1000; 83506, CST, USA), anti-HIF-1α (1:1000; ab2185, Abcam), anti-CDK9 (1:1000;2316,CST), anti-BRD4 (1:1000; ab128874, Abcam), anti-p38 (1:1000; 8690, CST), anti-Phospho-p38 (1:1000; 4511, CST), anti-JNK (1:1000; 9252, CST), anti-Phospho-JNK (1:1000; 4668, CST), anti-ERK1/2 (1:1000; 4695, CST), anti-Phospho-ERK1/2 (1:1000; AF1015, Affinity, China), or GAPDH (1:1000; MB001, Bioworld, USA). Subsequently, HRP-conjugated secondary anti-bodies including anti-rabbit and anti-mouse antibodies (Fcmacs, China) were incubated for 1 h. Blots were then visualized by a chemiluminescent imaging system (Tanon, China). The optical density of each lane was read using ImageJ (Bethesda, USA).

### Detection of intracellular Fe^2+^ amount

To detect intracellular Fe^2+^, FerroOrange (DojinDo, Japan) were used according to the manufacturer’s protocol. PDLFs were seeded on the confocal dishes and were washed with Hank’s balanced salt solution (HBSS) (Gibco, USA) to remove the residual reagents. Then cells were treated with 1 μmol/L FerroOrange with HBSS for 30 min at 37 °C. The cells were observed under a confocal laser scanning microscopy (Nikon A1, Japan).

### LIP measurement

The LIP was visualized using the fluorescent PG SK^38^. Briefly, after cells were treated with indicated stimuli, cells were trypsinized, collected, and resuspended at in 20 μM PG SK (Thermo Fisher, USA) in HBSS. Samples were incubated for 10 min in dark at 37 °C for 10 min. The sample was split into two aliquots with one treated with 2,2’bipyridyl (BIP) (Sigma, USA) for 15 min, and analyzed on flow cytometry (BD Bioscience, USA). The LIP (A.U.), which is equal to MFI_BIP_−MFI_NoBIP_, was normalized against the control samples to calculate the relative LIP.

### Immunofluorescence staining

PDLFs were treated with 8 mM butyrate for 24 h, fixed and blocked with 5% BSA (BioFroxx, China) and 0.01% Triton X-100 (BioFroxx, China) for 60 min, the primary and secondary antibodies (respectively A5695, ABclonal, 1:200; 83506, CST, 1:200; 5230-0427, KPL, 1:200 and 142902, Jackson ImmunoResearch, 1:200) were applied according to the manufacturer’s instruction. The interaction was analyzed by the confocal laser scanning microscopy (Nikon A1, Japan).

### Determination of ROS generation

Intracellular ROS in PDLFs was detected using ROS Assay Kit (Beyotime Biotech, China), PDLFs were seeded at a density of 2 × 10^5^ cells/well in six-well cell culture plate and treated with BA for 24 h, cells were using trypsin enzymic digestion and collecting cells, Cells were incubated with 10 mM DCFH-DA reagent for 30 min at 37 °C, then cells were washed in PBS and centrifuged three times. The cells were analyzed by flow cytometry (BD Bioscience, USA). The data were analyzed with FlowJo v.9.5.2 software (Tree Star).

### Lipid peroxidation and GSH assays

The generation of malondialdehyde (MDA) was used to measure lipid peroxidation according to the manufacturer’s protocol (Beyotime, China). Cells were harvested by trypsinization and cellular extracts were prepared by sonication in ice-cold buffer. After sonication, lysed cells were centrifuged at 10,000×*g* for 10 min to remove debris. The supernatant was subjected to the measurement of MDA levels. The protein concentration of each sample was determined by Nanodrop (Thermo Fisher Scientific, USA). The GSH and GSSG were determined using commercially available kits (Beyotime, China). All procedures completely complied with the manufacturer’s instructions.

### Statistical analysis

The variances between the groups that are being statistically compared were similar. All data are representative of at least three independent repeats if not otherwise stated. The letter *n* refers to the number of independently performed experiments representative of the data shown in the figures. Statistical analysis were evaluated by SPSS 20.0 and a two-sided test was applied, the raw data applying *t*-test was normally distributed. Data were expressed as means ± standard deviation (SD). Statistical significance was described as follows: *p < 0.05, **p < 0.01, ***p < 0.001.
